# A Survey of ECT Recipients, Family Members and Friends: Are the Self‐Reported Reasons for The Recipients' Problems Being Addressed?

**DOI:** 10.1111/inm.70187

**Published:** 2025-12-11

**Authors:** John Read, Sue Cunliffe, Sarah Hancock, Chris Harrop, Lucy Johnstone, Lisa Morrison

**Affiliations:** ^1^ Department of Psychology and Human Development University of East London London UK; ^2^ Independent Researcher London UK; ^3^ Ionic Injury Foundation USA; ^4^ Lisa Morrison Training and Consultancy London UK

## Abstract

Electroconvulsive therapy is administered, internationally, to approximately a million people annually. This study aimed to collect the self‐reported factors contributing to the problems for which ECT is prescribed and assess how well mental health services addressed those factors. An online survey was responded to by 858 ECT recipients and 286 family members and friends, from 44 countries. The majority (84%) of ECT recipients reported childhood adversities and, of those, 78% believed those adversities had contributed to the problems for which ECT was prescribed. Only 32% reported that mental health services had asked about their childhood adversities, and 30% that their adversities had been ‘therapeutically addressed’. 81% of ECT recipients reported at least one of 11 recent stressors. Of these, 67% believed their stressors had contributed to their problems. 34% reported being asked about their stressors and 21% felt that the stressors had been ‘therapeutically addressed’. Mental health professionals, including nurses, should ensure that patients are asked about the life events and circumstances they believe contributed to the difficulties for which ECT is being considered, and offered some help with them.

## 1 Introduction

Electroconvulsive therapy (ECT) involves administering electricity to the brain, under general anaesthesia, six to 12 times over several weeks, to produce tonic–clonic seizures. The clinical team usually involves mental health nurses, a psychiatrist, and an anaesthetist. A recent meta‐synthesis of 16 qualitative studies of patients' perspectives found that reported outcomes ranged from short‐lived improvements and long‐term benefits to no positive effects and damage to memory and other cognitive functions (Wells et al. 2020). A meta‐analysis found a spectrum of views about ECT among researchers, from ‘probably ineffective but certainly causes brain damage… through to those who think it is the most effective treatment in psychiatry and completely safe’ (UK ECT Review Group 2003).

Presumably this lack of consensus about efficacy and safety contributes to what an international review called ‘large variation between continent, countries and regions in utilization, rates and clinical practice’ (Leiknes et al. 2012). ‘Notable regional variation in receipt of ECT’ occurs within the US (Peltzman et al. 2020). Audits in England report 12‐fold (Read et al. 2018) and 47‐fold (Read et al. 2021) differences in usage between districts.

### 1.1 Psychosocial Causal Factors and Interventions

Psychosocial factors play an important role in the aetiology of depression, psychosis and other mental health issues (Madigan et al. 2023; Read and Moncrieff 2022; Read and Sanders 2022; Read and Dillon 2013; Remes et al. 2021; Varese et al. 2012). A Trauma‐Informed approach has become standard in many services in the USA and UK. It recognises the causal role of adverse events, and is based on a synthesis of findings from trauma studies, attachment theory and neuroscience. It integrates the causal roles of the mind, the body, relationships (particularly in early life) and the social world (Substance Abuse and Mental Health Services Administration 2014; Sweeney et al. 2016a). The original study on Adverse Childhood Experiences (ACEs) (Felitti et al. 1998) has been replicated many times (Madigan et al. 2023), and the role of a range of other adversities, relational and social, has also been recognised. This includes factors such as poverty, racism and domestic and neighbourhood violence. New approaches, such as the Power Threat Meaning Framework (Johnstone et al. 2018; Read and Harper 2022) also place adversities and traumas at the centre of their perspective, focussing on ‘what happened to you?’ rather than ‘what is wrong with you?’

Nevertheless, studies reveal that most users of psychiatric services, internationally, are neither asked about adversities and traumas (Cunningham et al. 2016; Neill and Read 2022; Nixon and Quinlan 2022; Read et al. 2018a), nor receive help to address these experiences when disclosed (Read et al. 2018b; Sweeney et al. 2016a).

An audit of 36 ECT information leaflets in England (Harrop et al. 2021) found that none made any mention of ‘psychosocial causes of depression, such as loss, abuse, poverty etc.’, but 21 (58%) of the leaflets referred to ‘biological causes of depression such as chemical imbalance, genetics etc.’, or framed problems as ‘illness’ or ‘disease’. Furthermore 28 (78%) included ‘Claims that ECT corrects biological causes of depression such as biochemical imbalance/activity, brain connectivity etc.’ An audit in Northern Ireland, Scotland and Wales found almost identical rates of claims about biological causes, and the correction thereof, but found that 48% did mention psychosocial causes (Read, Morrison, and Harrop 2023). Just over half of the ECT recipients (58%), and relatives/friends (53%), responding to the current study recall being told that depression is caused by ‘a chemical imbalance in the brain’ (Read, Harrop, et al. 2025).

This paper is one of a series summarising the results from a large‐scale survey of the views and experiences of ECT recipients and their relatives or friends (Morrison et al. 2025; Read, Harrop, et al. 2025; Read, Johnstone, Hancock, Harrop, et al. 2025; Read, Johnstone, Hancock, Morrison, et al. 2025). The current article focuses on whether participants felt that psychosocial causal factors, past and recent, had contributed to the difficulties for which ECT was prescribed, and if so, whether those factors had been asked about, and addressed, by mental health services.

## 2 Method

This study was approved by the University of East London's Ethics and Integrity Sub Committee (ETH2324‐0053). It used the same method as online surveys about other psychiatric treatments (Cartwright et al. 2016; Larsen‐Barr et al. 2018; Moncrieff et al. 2024; Read et al. 2006, 2014, 2017; Read, Lewis, et al. 2023; Read and Sacia 2020; Read and Williams 2019).

### 2.1 Instrument

A questionnaire was developed for the current study, based on the research literature and the experiences of the three members of the research team who have had ECT. The UK's largest mental health NGO, *Mind*, commented on a draft. The survey included quantitative questions with binary and multiple‐choice response options, and qualitative questions inviting written responses. Responses to survey questions about other aspects of ECT have been published elsewhere: information given to patients and family members about ECT (Read, Harrop, et al. 2025), efficacy (Read, Johnstone, Hancock, Harrop, et al. 2025), memory loss (Read, Johnstone, Hancock, Morrison, et al. 2025), other adverse effects (Read, Cunliffe, et al. 2025), and sex differences (Morrison et al. 2025).

Participants had to be at least 18 years old and either have had ECT (not in the past 4 weeks) or be ‘a friend or relative with an understanding of the impact of ECT’ on the person concerned.

The current paper reports on responses to the questions about: causal beliefs in general, the ten ACEs (Felitti et al. 1998), 11 stressors in the 6 months before ECT, how much the ACEs and stressors were believed to have contributed to the problems for which ECT had been prescribed, and whether these factors had been asked about, and addressed, by mental health services. Respondents were also asked whether they had received psychological therapy before ECT.

### 2.2 Procedure

The questionnaire was open, via Qualtrics, from January to September 2024, and disseminated on social media, including Instagram, Facebook, Twitter/X and YouTube. The researchers also contacted multiple individual and umbrella mental health organisations on six continents. For example, the 44 national group members of Mental Health Europe (www.mentalhealtheurope.org) were asked to disseminate the survey to their members. No translations of the survey were made.

### 2.3 Data Analysis

1211 responses answered at least some questions. There were 63 repeat responses (identified by IP address), 55 of which were deleted because the demographics and/or responses were similar. Twelve responses were deleted because of grossly discrepant responses (e.g., last ECT at age 16, first at 100 years), ‘straight‐lining’ on at least three questions (selecting the same option for lists of multiple items, e.g., ‘severe’ for all 27 side effects), being a recipient's nurse, or because more than one relative of the same patient had responded. This left 1144 for analysis. Of those, between 723 and 844 ECT recipients, and between 205 and 226 family/friends, responded to the various questions covered by this paper. The responses were analysed in terms of frequencies and percentages.

### 2.4 Patient and Public Involvement

The three members of the research team who have received ECT were involved in all aspects of the study, from design of the survey to manuscript preparation. The UK's largest mental health charity, *Mind*, commented on an early draft of the survey and subsequently distributed the survey via its ‘lived experience network’.

## 3 Results

### 3.1 Sample Characteristics

#### 3.1.1 Demographics

Seven countries provided at least 2% of the total respondents: USA (44%), UK (18%), Australia (10%), Canada (7%), Spain (3%), Ireland (2%) and New Zealand (2%). 37 other countries, provided up to 15 respondents. Most respondents were white (87% of recipients, 89% family/friends). Most were female (73% recipients, 68% family/friends). The average ages at the time of last ECT were 37 years (recipients) and 42 years (family/friends), ranging from 12 to 87.

#### 3.1.2 ECT

Most first and last courses of ECT occurred between 2010 and 2024 for both recipients (65% and 73% respectively) and relatives/friends (57% and 59%). The earliest was 1958. When asked to select one or more reasons ECT was given, 74% chose ‘Depression’, 17% ‘Psychosis/schizophrenia’, 15% ‘Bipolar disorder/mania’, 8% ‘Catatonia’, 13% ‘Other’ and 6% ‘Don't know’.

### 3.2 Biological Versus Psychosocial Causal Beliefs

Participants were asked: ‘To what extent do you think the problem for which ECT was prescribed (e.g., depression) was caused by biological factors (chemical imbalance, brain disorder, genetics etc.) vs. psychological/social factors (e.g., loneliness, stress, loss/bereavement, abuse, neglect, poverty, violence, discrimination etc)’, and given seven possible responses (e.g., ‘100% Biological’; ‘20% Biological – 80% Psychosocial’). About half the recipients (50.8%) and relatives/friends (47.8%) thought the causes of their problems were mostly psychosocial. About a third (29.0% recipients; 36.5% relatives/friends) thought they were mostly biological.

### 3.3 Childhood Adversities

Of the 735 ECT recipients who responded to the question about childhood adversities, 620 (84.4%) reported one or more of the ten ACEs, most frequently verbal abuse (53.9%) (see Table 1). 319 (43.6%) reported four or more ACEs.

**TABLE 1:** Childhood adversities (ACEs). ‘Did you experience any of the following before age 18?’^a^ (*n* = 735).

Parent or adult in your home ever swear at you, insult you, put you down	396 (53.9%)
Live with anyone depressed, mentally ill, or attempted suicide	338 (46.0%)
Feel that no one in your family loved you or thought you were special	316 (43.0%)
Experience unwanted sexual contact	286 (38.9%)
A parent or adult in your home ever hit, beat, kick, or physically hurt you	283 (38.5%)
Live with anyone who had a problem with drinking or using drugs	237 (32.2%)
Lose a parent through divorce, abandonment, death, or other reason	202 (27.5%)
Your parents or adults in your home ever hit, punch, beat, or threaten to harm each other	184 (25.0%)
Didn't have enough to eat, had to wear dirty clothes, no one to protect or take care of you	183 (24.9%)
Live with anyone who went to jail or prison	35 (4.8%)

^a^ Family/friends were not asked this question.

Of the 726 ECT recipients who answered, ‘Did any of those childhood experiences contribute to the problem(s) for which you were given ECT?’ 567 (78.1%) felt that they did contribute, to some extent (see Table 2).

**TABLE 2:** Did childhood adversities contribute to problems, and were they asked about and responded to?

‘Did any of those childhood experiences contribute to the problem(s) for which you were given ECT?’ (*n* = 726)	
A lot	254 (35.0%)
Somewhat	151 (20.8%)
A little	162 (22.3%)
Not at all/I had no negative childhood experiences	159 (21.9%)
‘Were you ever asked, by mental health staff/services, whether you had experienced any of those negative childhood experiences, before having ECT?’ (*n* = 732)	
Yes	231 (31.6%)
No	353 (48.2%)
Not sure	148 (20.2%)
‘Were any of those negative childhood experiences therapeutically addressed by mental health staff/services?’ (*n* = 606)	
Yes	183 (30.2%)
No	325 (53.6%)
Not sure	98 (16.2%)

Of the 732 responding to the question about being asked about childhood adversities by mental health staff/services, 353 (48.2%) said ‘No’, and 148 (20.2%) were ‘Unsure’ (see Table 2). Table 2 also shows that of the 606 who had experienced one or more childhood adversities and answered the question ‘Were any of those negative childhood experiences therapeutically addressed by mental health staff/services?’, 325 (53.6%) replied ‘No’.

### 3.4 Recent Stressors

Of the 735 ECT recipients who answered the question about 11 recent stressors, 602 (81.9%) had experienced at least one, most frequently ‘loneliness/isolation’ (see Table 3). Of the 218 family/friends who answered the question, 125 (57.3%) reported that the ECT recipient had encountered at least one recent stressor, most frequently, again, ‘loneliness/isolation’.

**TABLE 3:** Recent Stressors. ‘In the 6 months before most recent ECT did.’ you/X experience any of the following?

	Recipients (735)	Family/friends (218)
Loneliness/isolation	369 (50.2%)	72 (33.0%)
Other major negative life event	230 (31.3%)	56 (25.7%)
Coercive/emotional abuse	173 (23.5%)	40 (18.3%)
Other major loss	166 (22.6%)	38 (17.4%)
Poverty/unable to pay bills	113 (15.4%)	26 (11.9%)
Relationship breakup	108 (14.7%)	24 (11.0%)
Bullying at work	88 (12.0%)	17 (7.8%)
Death of a loved one	84 (11.4%)	28 (12.8%)
Domestic abuse	48 (6.5%)	15 (6.9%)
Rape/sexual assault	47 (6.4%)	13 (6.0%)
Other violence/abuse	46 (6.3%)	22 (10.1%)
Homelessness	30 (4.1%)	2 (0.9%)

Of the 692 recipients who answered the relevant question, 523 (75.6%) felt that recent stressors had contributed to the problem for which ECT was given. 59.7% felt that the stressors contributed ‘somewhat’ or ‘a lot’. Table 4 shows that the responses of family/friends were similar.

**TABLE 4:** Did recent stressors contribute to problems, and were they asked about and responded to?

	Recipients	Family/Friends (*n* = 175)
‘Did any of those experiences in the 6 months before ECT contribute to the problem(s) for which you were given ECT?’ (*n* = 692)		
A lot	265 (38.3%)	73 (41.7%)
Somewhat	148 (21.4%)	32 (18.3%)
A little	110 (15.9%)	19 (10.9%)
Not at all/I did not have any negative experiences	169 (24.4%)	51 (29.1%)
**‘**Were you ever asked, by mental health staff/services, about any of those experiences, before having ECT’ (*n* = 696)		
Yes	240 (34.5%)	^a^
No	300 (43.1%)	
Not sure	156 (22.4%)	
**‘**Were any of those experiences therapeutically addressed by mental health staff/services?’ (*n* = 621)		
Yes	154 (24.8%)	^a^
No	341 (54.9%)	
Not sure	126 (20.3%)	

^a^ Relatives/friends not asked these questions.

Of the 696 responding to the question about whether they were asked about recent stressors, 300 (43.1%) said ‘No’ (see Table 4). Table 4 also shows that of the 621 who had experienced recent stressors and answered the question ‘Were any of those experiences therapeutically addressed by mental health staff/services?’, 341 (54.9%) replied ‘No’.

### 3.5 ‘Before ECT Was Prescribed Did You/X Try Psychological Therapy/Counselling?’

Of the 844 recipients who answered the relevant question, 622 (73.7%) had tried psychological therapy/counselling, with a lower percentage (55.7%) reported by 271 family/friends.

### 3.6 Recency

Results suggest that clinical practice is improving over time. On all five clinical practice variables (asking about and addressing ACEs and recent stressors, and psychological therapy having been tried) the mean year of last ECT was significantly more recent for affirmative responses than for negative ones (all *p* < 0.001). For example, the average year for recent stressors being therapeutically addressed was 2016.2, compared to 2008.7 for those whose stressors had not been addressed (*t* = 6.25, df = 389.4, *p* < 0.001).

## 4 Discussion

### 4.1 Self‐Reported Causal Factors

Only about a third of ECT recipients (29.0%) thought the problems for which ECT had been prescribed were predominantly or exclusively biological.

Most recipients (84.4%) reported at least one ACE, and 43.4% reported four or more. A recent international meta‐analysis, of 206 studies and more than half a million people, found that an average of 60.1% of people report at least one ACE, and 16.1% report four or more (Madigan et al. 2023). The authors concluded:

Trauma‐informed practice requires having personnel who are sensitive to the impacts of adversity, recognise how the signs and symptoms of toxic stress manifest in individuals, integrate knowledge of ACEs and their impacts into their work practice, and can actively resist harm or re‐traumatization.

Most ECT recipients (78.1%) believed the ACEs had contributed to their problems. The participants had experienced very high levels of trauma in comparison to the general population (Madigan et al. 2023), and research has demonstrated that this constitutes a raised risk of mental health problems (Nelson et al. 2020; Read et al. 2001; Varese et al. 2012). It is reasonable therefore, to agree with the participants about the causal role of the ACEs in the distress for which ECT was prescribed. Similarly, most recipients (81.9%), and family/friends (57.3%), reported that one or more of the listed recent stressors had occurred in the 6 months before ECT, and most recipients (75.6%) and family/friends (70.9%) believed those stressors had contributed to the problems being treated by ECT.

These causal beliefs, of patients and non‐patients, are consistent with multiple previous findings that psychosocial causes of mental health problems are usually endorsed more than bio‐genetic factors, not only by recipients of mental health services/treatments (Carter et al. 2018; Dudley et al. 2009; Hansson et al. 2010; Read 2020; Read et al. 2014) but also by the public (Ahuvia et al. 2024; Angermeyer et al. 2014; Magaard et al. 2017; Pilkington 2013; Read et al. 2006, 2013).

An exception is the USA, where bio‐genetic causal beliefs are somewhat more popular (Pescosolido et al. 2010; Read et al. 2006, 2013). In the current study, recipients from the USA scored significantly more towards the biological end of the causes scale (Table 1) (mean = 4.44) than all other recipients combined (4.75) (*t* = 2.14, df = 718, *p* < 0.05). Even the USA mean was, however, on the psychosocial side of the midpoint (4).

### 4.2 Addressing the Self‐Reported Causal Factors

Only about a third of ECT recipients (31.2%) report being asked about any of the 10 ACEs, and only 30.2% of those who had experienced one or more of them thought that they had been ‘therapeutically addressed’ by mental health services. Similarly, only 34.5% had been asked about any of the 11 recent stressors. Even fewer (21.0%) of those who reported one or more of those stressors believed their stressors had been therapeutically addressed. This is consistent with studies that have found that the majority of users of mental health services are neither asked about adverse or traumatic events nor have them adequately addressed if disclosed (Agar et al. 2002; Cunningham et al. 2016; Neill and Read 2022; Nixon and Quinlan 2022; Read et al. 2018a, 2018b; Sweeney et al. 2016a, 2016b).

This failure to address psychosocial issues in the respondents' lives, while promoting a bio‐genetic model of aetiology and treatment, is consistent with the audits of ECT information leaflets (Harrop et al. 2021; Read, Morrison, and Harrop 2023), and what many of the participants had been told about causation (Read, Harrop, et al. 2025). The current survey found that 58% of recipients and 53% of family/friends were told ‘Depression is caused by a chemical imbalance in the brain’ and 42% and 41%, respectively, were told that ‘ECT corrects a chemical imbalance or other brain abnormality’ (Read, Harrop, et al. 2025).

We found that addressing childhood adversities and recent stressors seems to be improving over time, but the needs of many patients are clearly still not being met. When 21 women recipients of ECT in Norway were recently interviewed, the researchers (Coman and Bondevik 2024) reported:

A subgroup of nine participants described more negative experiences with ECT. A common factor for these participants was the experience of trauma that remained under‐treated.

We concur with the Norwegian researchers' conclusion that:

Educational modules for mental health care staff should include, besides knowledge on the methods' effectiveness, additional evidence about treatment recipients' subjective concerns and the relevance of trauma and recovery‐oriented care models.

### 4.3 Psychological Therapy

Even though the majority had tried psychological therapy, most had neither been asked about adversities nor had them therapeutically addressed. This suggests that some psychological therapists and counsellors are not working from a trauma‐informed perspective. Alternatively, the therapy or counselling may have been focused on matters unrelated to adversity and trauma. It is also worth noting that not all adversities are best addressed by psychological therapy.

Some of the causal factors mentioned by participants, such as loneliness, require practical or social interventions rather than therapy. For example, a recent study of over 15 000 older women found that widowhood, loneliness and social non‐participation were strongly predictive of depression, and recommended community interventions, not just counselling (Das et al. 2024).

The American Psychiatric Association (2025) makes no mention of the need for psychotherapy, or any other non‐medical treatment approaches, *before* ECT, in its recent 470‐page Task Force Report on ECT. It does state, however,

Psychotherapy should be considered after insufficient response to ECT to target ongoing psychosocial stressors or concomitant psychiatric disorders. (p. 283)

After an acute ECT course, individual, group or family psychotherapy can be a useful component of maintenance treatment for many patients. (p. 296)

This presents a challenge to the argument that ECT patients are not offered psychotherapy before ECT because they are too disturbed to benefit from it.

### 4.4 Adverse Effects

The importance of considering other treatment approaches becomes clearer when the adverse effects are considered. These include temporary and permanent memory loss (Read et al. 2019; Read and Moncrieff 2022; Rose et al. 2003; Sackeim et al. 2007; Shipwright and Murphy 2024) and major adverse cardiac events (Duma et al. 2019; Read 2024). In 2008, the United Nations declared:

It is of vital importance that ECT be administered only with the free and informed consent of the person concerned, including on the basis of information on the secondary effects and related risks such as heart complications, confusion, loss of memory and even death.

In 2023 the World Health Organisation and the United Nations stated: ‘People being offered ECT should be made aware of all its risks and potential short‐ and long‐term harmful effects, such as memory loss and brain damage’.

The four measures of memory problems used in the current survey produced rates from 61% to 84%. For 65% of those experiencing anterograde amnesia, and 81% of those reporting retrograde amnesia, the problems lasted at least 3 years. (Read, Johnstone, Hancock, Morrison, et al. 2025).

### 4.5 Limitations

#### 4.5.1 Sample Biases

Sample bias towards people with a *negative* opinion of ECT may have occurred because of the dissemination of the survey on social media by the researchers, some of whom have critiqued ECT in research journals, books and online. To minimise this, social media posts included phrases like ‘Positive, mixed and negative experiences are all equally valued’.

Sample bias in favour of people with a *positive* view of ECT may also have occurred, in four ways. First, people for whom ECT had not alleviated the severe depression for which it is often prescribed might be uninterested in, or unable to complete, surveys. Second, people whose suicidality was not alleviated by ECT, and who killed themselves, could not participate. Third, patients who died during or soon after treatment due to cerebral or cardiovascular events (Duma et al. 2019; Read 2024) did not participate. Fourth, some of those in whom ECT caused severe cognitive damage may have been unable to participate.

Despite the subjectivity involved in patient‐reported outcome measures (PROMs) they are increasingly used in clinical practice (Carfora et al. 2022). Some researchers suggest that patient satisfaction surveys tend to be biased towards positive reports of services and treatments (Dunsch et al. 2018).

A study of internet‐based surveys in the English NHS found that ‘patients’ website ratings of hospitals and more conventional measures of patient experience from large random surveys are significantly correlated (Greaves et al. 2012). It concluded ‘Our findings add to the increasingly persuasive literature promoting the notion that one needs to view safety, quality and service delivery through a number of lenses to get an accurate picture’.

#### 4.5.2 Other Limitations

An important caveat is that self‐report relies on memory, which may be inaccurate due to the passing of time (sometimes many years) and/or to the effects of ECT. It is possible, for example, some participants were asked about traumatic events but they have no recall of being asked; or that their statements about difficult life events were inaccurate (either under‐reported or over‐reported). Additionally, it is not possible to ascertain whether the reported psychosocial contexts were, or were not, causal factors in the subsequent mental health conditions, only that participants and their relatives believed that they were.

It is possible that some people who did not meet the inclusion criteria may have completed the survey. The lack of remuneration for doing so may have reduced that possibility.

This survey was limited to people who can read English, because survey translations were not available. Consequently, survey participants are primarily from English‐speaking countries, predominantly in North America, Europe and Australasia.

## 5 Conclusions

These reports suggest that several hundred ECT recipients, family members and friends, believe that both childhood adversities and recent stressors had played a role in causing the problems for which ECT was being prescribed. These psychosocial causal factors are, according to recipients and those who know them, frequently not being addressed, or even asked about. Survey respondents indicate that for some people ECT is being prescribed without trying psychological therapy first.

## 6 Relevance for Clinical Practice

We recommend that all mental health staff, including nurses, ensure that people being prescribed ECT have been asked about past and recent adversities, and that any disclosed adversities are addressed. They should ensure that people being prescribed ECT have first been offered psychological therapy, along with other forms of support appropriate to their past or current psychosocial problems and needs.

## Author Contributions

All authors listed meet the authorship criteria according to the latest guidelines of the International Committee of Medical Journal Editors, and are in agreement with the manuscript.

## Funding

The authors have nothing to report.

## Conflicts of Interest

John Read has been a paid expert witness in several ECT legal cases in the USA, New Zealand and Canada. No other authors have any conflicts of interest.

## Data Availability

The data that support the findings of this study are available on request from the corresponding author. The data are not publicly available due to privacy or ethical restrictions.
